# Thoracic Endovascular Aortic Repair for Aberrant Subclavian Artery and Stanford Type B Aortic Intramural Hematoma

**DOI:** 10.3389/fsurg.2021.813970

**Published:** 2022-02-11

**Authors:** Xia Xu, Daoquan Wang, Ningxin Hou, Hongmin Zhou, Jun Li, Liang Tian

**Affiliations:** ^1^Department of Cardiothoracic and Vascular Surgery, Tongji Medical College, Tongji Hospital, Huazhong University of Science and Technology, Wuhan, China; ^2^Department of Neurosurgery, Taikang Tongji (Wuhan) Hospital, Wuhan, China

**Keywords:** aberrant subclavian artery, Kommerell's diverticulum, aortic intramural hematoma, endovascular repair, penetrating atherosclerotic ulcer, ulcer-like projection

## Abstract

**Objectives:**

To evaluate the in-hospital and later outcomes of thoracic endovascular aortic repair (TEVAR) for type B intramural hematoma (TBIMH) combined with an aberrant subclavian artery (aSCA).

**Methods:**

In the period from January 2014 to December 2020, 12 patients diagnosed with TBIMH combined with aSCA and treated by TEVAR were enrolled in this retrospective cohort study, including 11 patients with the aberrant right subclavian artery (ARSA) and 1 with an aberrant left subclavian artery (ALSA). A handmade fenestrated stent-graft or chimney stent or hybrid repair was performed when the proximal landing zone was not enough.

**Results:**

The mean age of all the patients was 59.2 ± 7.6 years, and 66.7% of patients were men. There were 4 patients with Kommerell's diverticulum (KD). The procedures in all 12 patients were technically successful. There was one case each of postoperative delirium, renal impairment, and type IV endoleak after TEVAR. During follow-up, 1 patient died of acute pancreatitis 7 months after TEVAR. The overall survival at 1, 3, and 5 years for the patients was 90.9, 90.9, and 90.9%, respectively. KD was excluded in 2 patients, and the handmade fenestrated stent-graft was applied in the other 2 patients to preserve the blood flow of the aSCA. No neurological complications developed and no progression of KD was observed during the follow-up.

**Conclusion:**

Thoracic endovascular aortic repair for patients with aSCA and TBIMH is promising. When KD was combined, we could exclude KD or preserve the blood flow of aSCA with regular follow-up for the diverticulum according to the size of the KD.

## Introduction

Aberrant subclavian artery (aSCA) is a common congenital anomaly of the aortic arch with two typical subtypes: left-sided aortic arch with an aberrant right subclavian artery (ARSA) and right-sided aortic arch with an aberrant left subclavian artery (ALSA) (1). ARSA is relatively common, with an incidence of 0.5–1.8%, while ALSA is less common, with an incidence of 0.05% (2). It is reported about 20–60% of patients with aSCA have an associated Kommerell's diverticulum (KD), an aneurysmal aortic dilatation at the origin of the aSCA, which rarely causes dysphagia or dyspnea but may progress to aortic dissection (AD) or rupture (3, 4). Recent studies suggest that aSCA is associated with aortic pathology, such as aortic aneurysms and AD (1, 5). What is more, the presence of aSCA usually complicates the treatment strategy in aortic lesions.

Acute aortic syndromes comprise a constellation of lethal medical entities, such as acute AD, aortic intramural hematoma (IMH), and penetrating atherosclerotic ulcer (PAU) (6). Lesions that do not involve the ascending aorta are classified as Stanford type B, of which most can be treated by thoracic endovascular aortic repair (TEVAR) with less trauma and fewer complications (6). However, when combined with type B acute aortic syndromes, the aSCA may affect the proximal landing zone (PLZ) of the stent and limit the application of TEVAR.

At present, there are some small-sample studies on TEVAR for type B aortic dissection (TBAD) and aSCA (7, 8), while only a small number of cases of type B intramural hematoma (TBIMH) and aSCA have been reported (9). Therefore, we conducted this retrospective study to evaluate the in-hospital and later outcomes of TEVAR for TBIMH combined with aSCA.

## Materials and Methods

### Study Design and Patients

**Figure 1:**
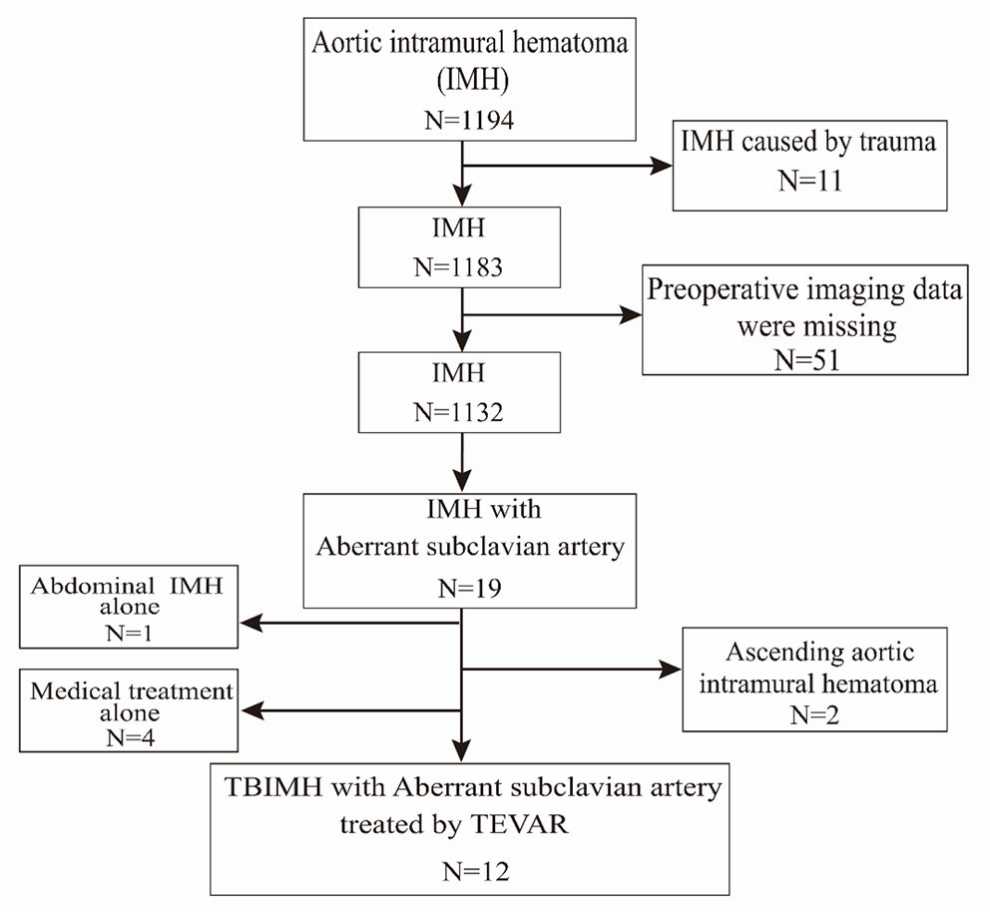
Flowchart of patients screened from electronic medical records. TEVAR, thoracic endovascular aortic repair.

We retrospectively collected data on patients with TBIMH and aSCA from January 2014 to December 2020 in Tongji Hospital of Tongji Medical College of Huazhong University of Science and Technology. The flowchart of screening patients from electronic medical records is shown in Figure 1. There were 1,194 consecutive patients with IMH admitted to our department during the 7 year period, of whom 19 patients had aSCA, including 18 with left-sided aortic arch and ARSA and 1 with right-sided aortic arch and ALSA. The ratio of aSCA to spontaneous IMH occurrence was 1.7% (excluding patients with traumatic IMH and missing imaging data). After further exclusion of patients with Stanford type A IMH, abdominal IMH alone, and medical treatment alone (Figure 1), 12 patients with TBIMH and aSCA treated by TEVAR were enrolled in this study.

The Ethics Committee of Tongji Hospital of Tongji Medical College of Huazhong University of Science and Technology approved this retrospective cohort study and individual patient consent was waived.

### Definition

**Figure 2:**
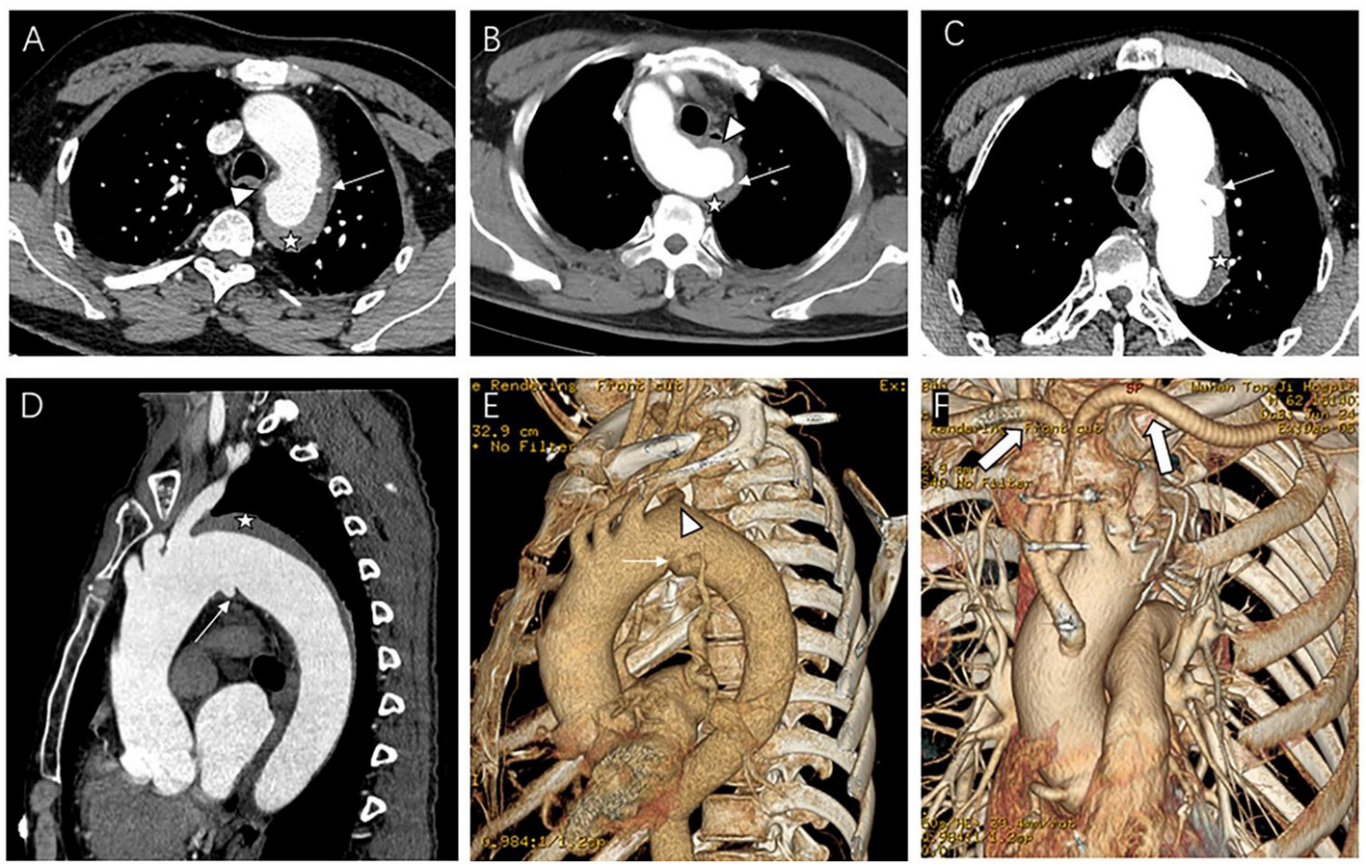
Contrast-enhanced CT angiography (CTA) image of the aberrant subclavian artery patients with aortic intramural hematoma (IMH) and penetrating atherosclerotic ulcer (PAU) or ulcer-like projection (ULP). **(A)** Patient with PAU (arrow) located near the aberrant right subclavian artery (ARSA) ostium (arrowhead) and IMH (star); **(B)** Patient with aberrant light subclavian artery and IMH (star), the PAU (arrow) located on the Kommerell's diverticulum (KD) (arrowhead). **(C–F)** Patient with ULP (thin arrow) located near the ARSA ostium (arrowhead) and IMH (star) underwent hybrid repair, thick arrow showing the artificial blood vessel from ascending aorta to subclavian arteries.

**Figure 3:**
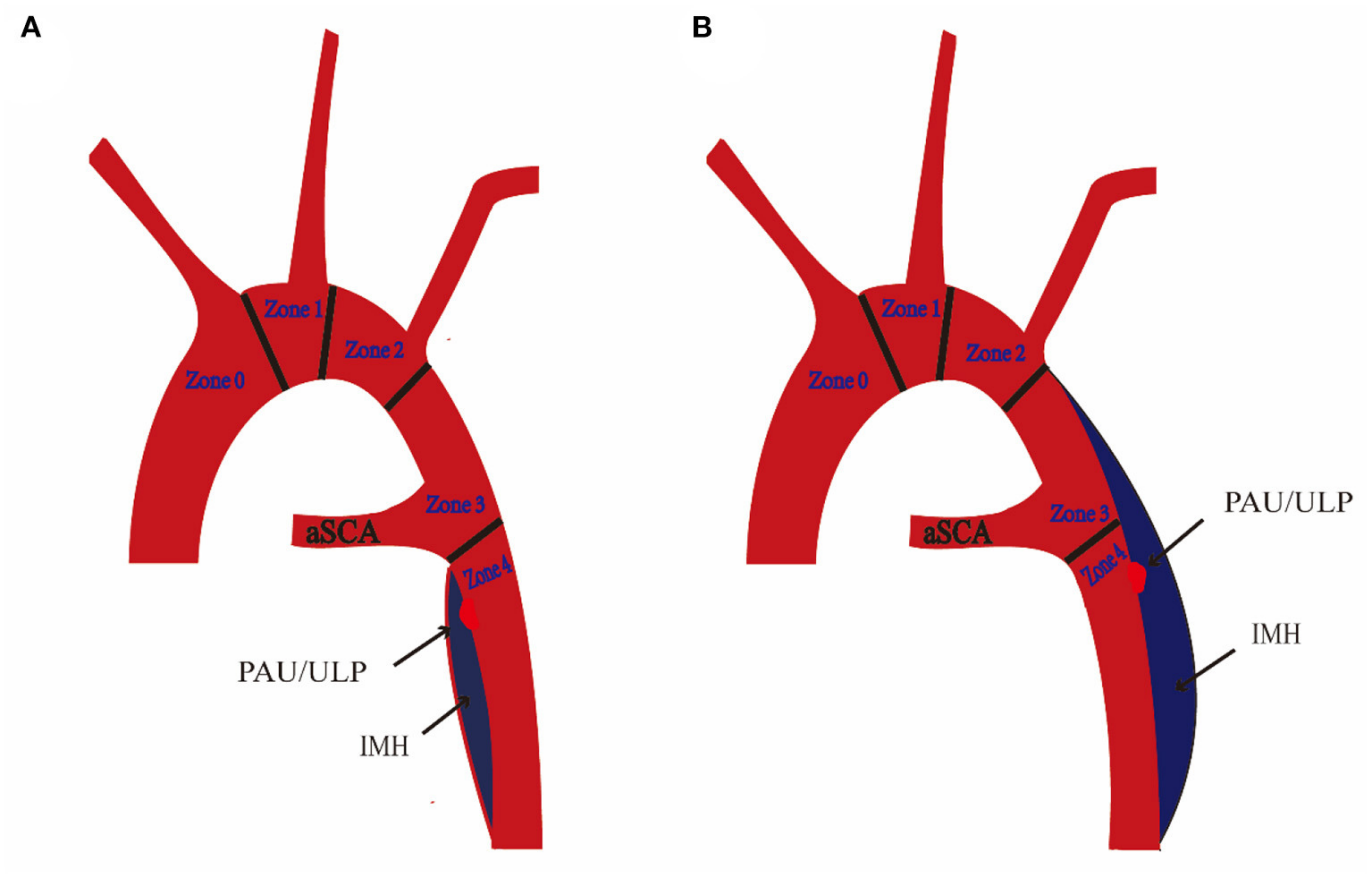
Schematic diagram of aberrant subclavian artery (aSCA) and type B intramural hematoma (TBIMH). **(A)** Proximal hematoma extending distal to the ostium of aSCA. **(B)** Proximal hematoma extending distal to the ostium of the left subclavian artery (left-sided aortic arch) or right subclavian artery (right-sided aortic arch). IMH, aortic intramural hematoma; PAU, penetrating atherosclerotic ulcer; ULP, ulcer-like projection.

We used a contrast-enhanced CT angiogram (CTA) image to define IMH, PAU, and ulcer-like projection (ULP). The latter two were often accompanied by IMH and considered to be risk factors for poor prognosis in IMH (10). TBIMH was defined as a hematoma involving the descending thoracic aorta larger than 5 mm in diameter and consisting of a circular or crescent-shaped thickening around the aortic wall, with no evidence of blood flow between the lumen and the aortic wall (11). PAU was defined as an aortic atherosclerotic lesion in the internal elastic lamina penetrating the media, and ULP was defined as a localized blood-filled pouch with obvious communication with the true lumen (10). KD was defined as a dilatation of the root of the aSCA more than 1.5 times the size of the distal subclavian artery (12). Figure 2 shows these different entities. The aortic zone was widely applied in patients with normal aortic arch morphology according to the anatomical range of the three branches, and Zone 3 extended from distal to the LSA to the proximal descending thoracic aorta (13). Since there was a fourth branch at the beginning of the descending aorta in patients with aSCA, we named the area between the two subclavian arteries Zone 3, according to the previous nomenclature (Figure 3).

### Management

All diagnoses were confirmed by the CTA examination on admission, and the origin and extent of the hematoma, the maximal hematoma thickness measured near pulmonary artery bifurcation level, the location of PAU or ULP, and the diameter at the original of KD were recorded.

Patients were closely monitored and heart rate and blood pressure were controlled as well as pain in the intensive care unit (ICU) before TEVAR. Indications for TEVAR were PAU or ULP in the CTA image, hematoma thickness >10 mm, persistent pain, and uncontrolled hypertension (10, 14).

In addition to the routine CTA review in the hospital, all the patients except those with renal insufficiency were examined by CTA at 3 months, 6 months, and annually after discharge. The mean follow-up time was 3.7 ± 1.8 years, and one patient was lost to follow-up after discharge.

### TEVAR Procedures

All TEVAR procedures were carried out in the hybrid operating room. Except for one patient with severe chronic obstructive pulmonary disease who was treated with local anesthesia and intravenous opiates, others received general anesthesia. The femoral artery approach was used in all patients. The aim of TEVAR was to (1) block PAU or ULP; (2) exclude the hematoma as much as possible but avoid covering large intercostal arteries. The management strategy of KD is controversial, and in general, surgical treatment is not recommended for asymptomatic KD (15). The specific threshold (usually 3 cm) for treating asymptomatic KD was based on the surgeon's estimation of aneurysm size and previous experience (4).

**Figure 4:**
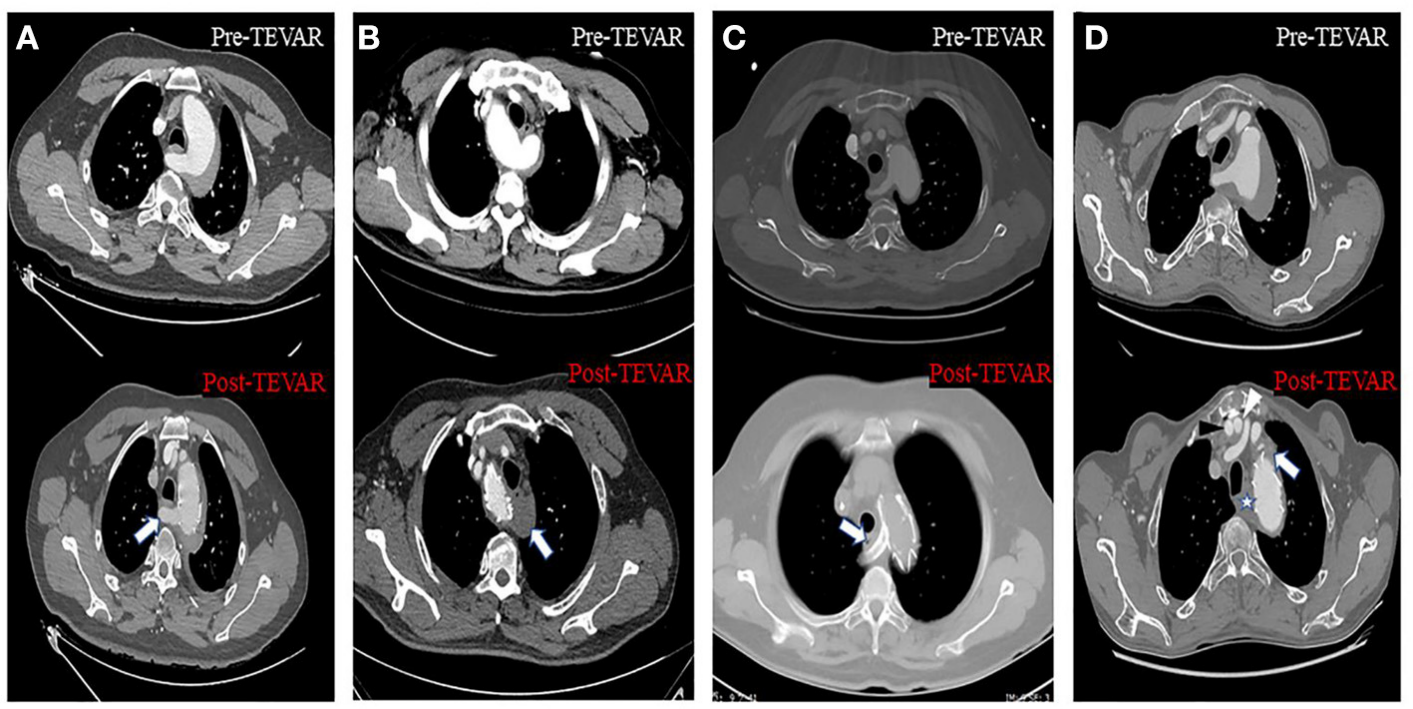
Axial CT images about four different approaches to solve the inadequate proximal landing zone when TEVAR was performed. **(A)** Handmade fenestrated stent graft: arrow showed that the blood flow of the aberrant right subclavian artery (ARSA) was preserved. **(B)** Aberrant left subclavian artery (ALSA) was covered without non-revascularization, and arrow showed the thrombosis at the origin of the ALSA. **(C)** Chimney stent technology: arrow showed the ARSA was patent after the chimney stent was implanted. **(D)** Hybrid repair: black and white arrowhead showed the artificial blood vessel from aorta to right and left subclavian artery, respectively; star and arrow showed the thrombosis at the origin of the ARSA and left subclavian artery (LSA), respectively.

For patients who needed reconstructive blood flow in the subclavian arteries, a chimney stent or handmade fenestrated stent-graft or hybrid repair was performed (Figure 4). The chimney stent implanted was Viabahn (W. L. Gore, Flagstaff, AZ, USA). We created fenestrations on Valiant (Medtronic, Minneapolis, MN, USA) or Relay (Bolton Medical, Sunrise, FL, USA) stents according to the preoperative CTA image and the aortogram during the procedure to preserve blood flow of the associated artery. The hybrid repair was performed by establishing the bypass from the ascending aorta to the subclavian arteries without cardiopulmonary bypass. In all the patients, the maximal oversize of the stent-graft was not more than 10%, and systolic blood pressure was kept below 100 mmHg during the whole procedure.

### Statistical Analysis

Statistical analysis was conducted with R software (version 3.6.1; R Foundation, Vienna, Austria). Categorical variables were summarized as numbers and percentages. Continuous variables were described as mean ± SD and range. Survival analysis was performed using the Kaplan–Meier method.

## Results

### Clinical Characteristics

The study population consisted of 11 patients with left-sided aortic arch and ARSA and 1 with right-sided aortic arch and ALSA (Table 1). The mean age of the 12 patients was 59.2 ± 7.3 years and 66.7% of patients were men. The most common comorbidity was hypertension (83.3%). Most patients had symptoms of chest pain (11/12, 91.7%), and no patients had chronic dysphagia and dyspnea.

**Table 1:** Clinical characteristics in the 12 patients with aberrant subclavian artery.

**Features**	**Number or mean (SD)**	**Range or %**
Age, year	59.2 (7.6)	49–70
Sex, male	8	66.7
Body mass index, kg/m^2^	24.6 (3.2)	19.0–30.5
Smoking	2	16.7
**Comorbidities**		
Hypertension	10	83.3
Hyperlipidemia	3	25.0
Chronic heart failure	3	25.0
Chronic obstructive pulmonary disease	2	16.7
Cerebrovascular disease	1	8.3
**Symptom**		
Chest and back pain	6	50.0
Chest pain	5	41.7
Chest tightness	1	8.3
**Aberrant subclavian artery**		
Left-sided aortic arch with ARSA	11	91.7
Right-sided aortic arch with ALSA	1	8.3

*Continuous variables were described as mean ± SD and range. Categorical variables were summarized as number and percentage. ARSA, aberrant right subclavian artery (ARSA); ALSA, aberrant left subclavian artery*.

### CTA Parameters on Admission

In the initial CTA image, the proximal hematoma extended to zone 3 in half of the patients, and the distal hematoma extended beyond the diaphragm in 9 (75.0%) patients (Table 2). IMH-associated lesions were observed in 8 patients, including PAU in 6 patients and ULP in 2 patients. Pleural effusion or pericardial effusion existed in a few patients, and the amount was small (Table 2). There were four patients with KD, of which the maximal diameter at the base ranged from 16.2 to 24.5 mm. The maximal hematoma thickness measured at the near pulmonary artery bifurcation level ranged from 5.5 to 13.9 mm.

**Table 2:** Image data of initial contrast-enhanced CT angiogram (CTA).

**Patient**	**Aberrant subclavian artery**	**Extent of the hematoma**	**Associated PAU or ULP**	**Pleural effusion/Pericardial effusion**	**KD/Diameter at the original (mm)**	**Maximal Hematoma Thickness (mm)**
1	ARSA	Zone 4/Below the diaphragm	PAU	–/–	Yes/16.2	13.3
2	ARSA	Zone 3/Below the diaphragm	PAU	–/–	Yes/20.5	7.3
3	ARSA	Zone 3/Below the diaphragm	PAU	–/–	–	6.2
4	ARSA	Zone 4/Below the diaphragm	ULP	–/–	–	5.8
5	ARSA	Zone 4/Below the diaphragm	–	–/–	–	10.6
6	ARSA	Zone 3/Below the diaphragm	–	small/–	–	13.9
7	ARSA	Zone 3/Above the diaphragm	ULP	–/–	–	5.7
8	ARSA	Zone 3/Below the diaphragm	–	small/–	–	10.1
9	ARSA	Zone 4/Above the diaphragm	PAU	–/–	–	5.6
10	ARSA	Zone 4/Below the diaphragm	PAU	small /small	Yes/20.2	10.3
11	ARSA	Zone 3/Below the diaphragm	–	small/–	–	8.7
12	ALSA	Zone 4/Above the diaphragm	PAU	–/–	Yes/24.5	5.5

*ARSA, aberrant right subclavian artery (ARSA); ALSA, aberrant left subclavian artery; PAU, penetrating atherosclerotic ulcer; ULP, ulcer-like projection; KD, Kommerell's diverticulum*.

### Surgical Data

The TEVAR procedures in all 12 patients were successful, and all the PAU or ULP were treated. Most patients underwent TEVAR within 3 days from admission (Table 3), except one who was treated 2 weeks after admission for refractory hypertension.

**Table 3:** Details about thoracic endovascular aortic repair (TEVAR).

**Patient**	**Time from admission to TEVAR (days)**	**Dominant vertebral artery**	**Distance from PAU/ULP to aSCA (mm)**	**Adjunctive procedures**	**Anesthesia**	**Number of stents**	**Operation time (min)**
1	1	LVA	18.8	Fenestrated stent for ARSA	General anesthesia	2	171
2	1	LVA	23.7	Fenestrated stent for ARSA	General anesthesia	2	105
3	3	Equal flow	13.5	Fenestrated stent for ARSA	General anesthesia	1	100
4	2	LVA	11.2	Chimney stent for ARSA	General anesthesia	2	217
5	3	RVA	–	Fenestrated stent for ARSA	General anesthesia	2	153
6	3	Equal flow	–	Fenestrated stent for ARSA	General anesthesia	2	158
7	14	LVA	Near the ARSA ostium	Bypass from ascending aorta to two subclavian arteries	General anesthesia	1	387
8	1	LVA	–	Fenestrated stent for ARSA	General anesthesia	2	148
9	1	LVA	15.0	Fenestrated stent for ARSA	General anesthesia	1	100
10	3	LVA	Near the ARSA ostium	Covered ARSA, non-revascularization	Local anesthesia	1	74
11	1	LVA	–	Fenestrated stent for ARSA	General anesthesia	2	103
12^†^	3	RVA	On the Kommerell's diverticulum	Covered ALSA, non-revascularization	General anesthesia	1	94

*TEVAR, thoracic endovascular aortic repair; ARSA, aberrant right subclavian artery; ALSA, aberrant left subclavian artery; PAU, penetrating atherosclerotic ulcer; ULP, ulcer-like projection; aSCA, aberrant subclavian artery; LVA, left vertebral artery*.

† *Patient with right-sided aortic arch and ALSA*.

In terms of surgical details, the dominant vertebral artery was analyzed before TEVAR and dominant LVA was the most common (Table 3). The distance from PAU/ULP to aSCA was within 20.0 mm in most patients, and in 3 patients, PAU/ULP was located near the aSCA. When the proximal landing zone was not large enough, we reconstructed the blood flow of aSCA in 10 patients: 8 patients had an implanted handmade fenestrated stent-graft, 1 patient had an implanted chimney stent for ARSA, and 1 patient underwent a hybrid technique with bypass from the ascending aorta to two subclavian arteries. The aSCA was intentionally covered in another 2 patients. In patients with hematoma extending below the diaphragm, most had two stents implanted, while all patients with hematoma extending above the diaphragm had one implanted stent (Table 3).

### In-Hospital and Follow-Up Outcomes

There was one patient with postoperative delirium, and it was controlled after using haloperidol (Table 4). One patient with normal renal function had renal impairment 8 days after TEVAR, and the estimated glomerular filtration rate (eGFR) declined to 53 from 92 ml/min/1.73 m^2^. Type IV endoleak appeared in one patient in the routine review of CTA after TEVAR. All patients were successfully discharged.

**Table 4:** In-hospital and follow-up outcomes.

**Patient**	**In-hospital outcome/time after TEVAR**	**Later outcome/time after TEVAR**	**Later survival /Time (years)**	**CTA follow-up IMH/KD**
1	Postoperative Delirium/4 days	Type II endoleak/9 months	Alive/3.66	Completely absorbed, no progression of KD
2	–		Alive/3.74	Completely absorbed, no progression of KD
3	Renal function was impaired/8 days	Renal function was not recovered	Alive/2.80	Partially absorbed
4	–	–	Alive/2.76	Completely absorbed
5	–	Death caused by acute pancreatitis/6 months	Death/0.64	Completely absorbed
6	–	–	Lost to follow-up	–
7	–	–	Alive/7.07	Completely absorbed
8	–	–	Alive/6.61	Completely absorbed
9	–	–	Alive/3.90	Completely absorbed
10	–	–	Alive/3.97	Completely absorbed
11	Type IV endoleak/7 days	Type IV endoleak disappeared	Alive/3.13	Completely absorbed
12	–	–	Alive/2.38	Completely absorbed

*TEVAR, thoracic endovascular aortic repair; CTA, contrast-enhanced computed tomography angiogram; KD, Kommerell's diverticulum*.

**Figure 5:**
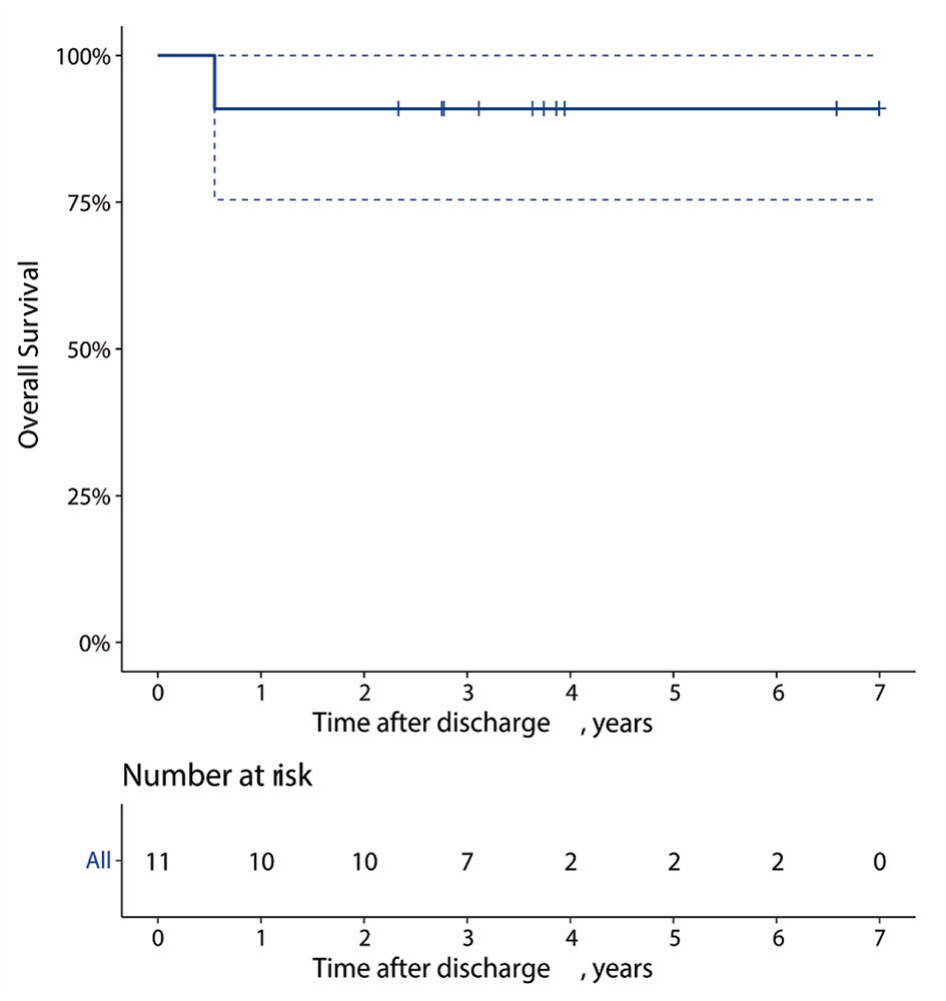
Kaplan–Meier curve of 7-year survival from all-cause mortality in the patients.

One patient was lost to follow-up, and one died of acute pancreatitis 7 months after TEVAR. The overall survival at 1, 3, and 5 years for the 11 patients was 90.9, 90.9, and 90.9%, respectively (Figure 5). In terms of follow-up of CTA, one patient had type II endoleak 9 months after TEVAR, and a type IV endoleak that arose in one patient during hospitalization disappeared (Table 4). In recent CTA images, the hematoma was completely absorbed in 10 patients. In one patient, renal function did not meet the threshold for CTA; the most recent CTA image of the patient was recorded 7 days after TEVAR during hospitalization, and the hematoma was reduced from 6.2 to 4.1 mm (Table 4). Meanwhile, the KD of two patients was not covered by TEVAR and showed no progression on follow-up (Table 4).

## Discussion

The simultaneous occurrence of aSCA and IMH is an extremely rare condition, with only a few cases up to now (9, 16, 17); ALSA with IMH has almost never been reported. The study of Magee et al. suggested an incidence (9.6%) of AD or aortic aneurysm in 312 patients with aSCA (1). Shalhub et al. suggested that flow hemodynamics were changed in patients with the variant arch anatomy and were associated with AD (18). These studies preliminarily indicated chronic aortic injury existed in patients with an aortic arch variation. Similar to AD, IMH is a lesion of the aortic wall and can progress to direct rupture or aneurysmal dilation (19). In this study, 1.7% (19/1,132) of IMH patients had aSCA; however, it could not be further inferred if there is a correlation between aortic arch variation and IMH.

Thoracic endovascular aortic repair is currently recommended for treating the complex TBIMH, such as persistent or recurrent pain, uncontrolled hypertension despite aggressive medical therapy, signs of aortic rupture, and other dangerous imaging findings, of which PAU or ULP indicate a poor prognosis (6, 19, 20). In TBIMH patients with a normal aortic arch, the stent-graft is usually anchored distal to the LSA. However, in TBIMH patients with aSCA, the PLZ is often affected by the aSCA. On the other hand, how to manage the possible KD that might accompany aSCA is also a complex issue.

Aortic replacement or a hybrid technique has been previously reported for aSCA patients with TBAD (21, 22). However, there is no consensus on the management of aSCA patients with type B aortic syndrome because there are too few cases. Because of the inevitable larger damage and slower recovery with conventional surgery, some researchers have been trying to address these complex lesions with emerging endovascular technology, and the results have shown favorable early and mid-term outcomes (7–9). Although these three studies had only 33 patients (ARSA with 29 TBAD and 4 TBIMH), the approaches to deal with the lack of PLZ varied, such as covering ARSA, ARSA revascularization, and LSA revascularization (7–9). The result was satisfactory, and only a few patients had transient symptoms of limb ischemia, although the ARSA was covered in most cases in these studies. These results seem to be inconsistent with the practice guideline from the Society of Vascular Surgery (23), which suggests preoperative revascularization of the left subclavian artery in patients who needed TEVAR with left subclavian artery coverage. However, subsequent studies have shown that left subclavian artery coverage does not increase the risk of spinal cord ischemia and cerebrovascular accidents when the dominant vertebral artery is preserved (24, 25). In general, it is controversial whether to perform revascularization or coverage of aSCA in these complex conditions. The surgeons in our center favor the preserving blood flow of the aSCA, and only two patients with KD underwent aSCA coverage without revascularization.

The common way to reconstruct blood flow of ARSA is ARSA periscope and single-branched stent-graft, as in previous studies (7–9), and Fang et al. discussed in detail the characteristics of various approaches to blood flow reconstruction (9). However, it was not clear which one was the most advantageous because of the limitations of sample size and follow-up time, and usually the most suitable approach was based on the characteristics of patients. Compared with chimney stent or single-branched stent graft, handmade fenestration stent cost less and could be rapidly implemented in emergency TEVAR, which was shown in our results that the time to implant chimney stent was longer than that to implant the handmade fenestration stent. Because of the unsatisfactory results reported in patients with TBAD undergoing hybrid repair, such as relatively higher mortality and higher incidence of type I endoleak (26, 27), we usually perform it when the stent needs to be anchored in Zone 2 and there is not enough distance between the subclavian arteries.

Generally, KD patients with symptoms of esophageal or tracheal obstruction or an orifice diameter beyond 30 mm need intervention, such as surgery, hybrid repair, and total TEVAR (28). Recent research by Hale et al. highlighted the rationale for conservative management of asymptomatic KD and questioned the increase in KD over time (29). There were four patients with KD in our study, and no one met the above intervention criteria. KD was excluded in two patients, including one with ARSA and one with ALSA, and a handmade fenestrated stent-graft was applied in the other two patients to preserve the blood flow of ARSA. No neurological complications developed in the two patients during follow-up because the dominant vertebral artery was preserved. There was no progression of KD in the latter two patients. These results suggested that when the diameter of the KD is large, the 30-mm threshold does not need to be reached and it might be addressed concurrently without the additional operative risks in these complex lesions. When the diameter of the KD is small, preservation of the blood flow of aSCA and regular follow-up for the diverticulum is reasonable as opposed to immediate closure.

Several limitations existed in this study. First, it was an observational and retrospective study with only 12 patients. Second, the follow-up was too short to fully reflect the later prognosis. Third, the TEVAR procedures were performed by different surgeons in our center, and because of the large time span of case inclusion and lack of established standards, their understanding of the specific revascularization method and the threshold to exclude KD may be inconsistent, leading to the inevitable bias in the results.

Fourth, more studies are needed to verify the rationality of excluding the KD with its diameter <30 mm in patients with these lesions.

## Conclusion

Thoracic endovascular aortic repair for patients with aSCA and TBIMH is promising. When combined with KD, we could exclude KD or preserve the blood flow of aSCA with regular follow-up for the diverticulum according to the size of the KD.

## Data Availability Statement

The raw data supporting the conclusions of this article will be made available by the authors, without undue reservation.

## Ethics Statement

The studies involving human participants were reviewed and approved by the Ethics Committee of Tongji Hospital of Tongji Medical College of Huazhong University of Science and Technology. Written informed consent for participation was not required for this study in accordance with the national legislation and the institutional requirements.

## Author Contributions

LT and JL were involved in the conceptualization, study design, and completed the writing—review and editing. HZ contributed to the project administration and supervision. DW contributed to the review of computed tomography data and visualization. NH collected the data. XX performed the statistical analysis and wrote the manuscript draft. All authors contributed to the article and approved the submitted version.

## Conflict of Interest

The authors declare that the research was conducted in the absence of any commercial or financial relationships that could be construed as a potential conflict of interest.

## Publisher's Note

All claims expressed in this article are solely those of the authors and do not necessarily represent those of their affiliated organizations, or those of the publisher, the editors and the reviewers. Any product that may be evaluated in this article, or claim that may be made by its manufacturer, is not guaranteed or endorsed by the publisher.

## Abbreviations

aSCA: aberrant subclavian artery
ARSA: aberrant right subclavian artery
ALSA: aberrant left subclavian artery
KD: Kommerell's diverticulum
AD: aortic dissection
IMH: aortic intramural hematoma
PAU: penetrating atherosclerotic ulcer
ULP: ulcer-like projection
TEVAR: thoracic endovascular aortic repair
PLZ: proximal landing zone
TBAD: type B aortic dissection
TBIMH: type B intramural hematoma
CTA: contrast-enhanced CT angiogram.
